# Oral administration of *Lactobacillus gasseri* SBT2055 is effective in preventing *Porphyromonas gingivalis*-accelerated periodontal disease

**DOI:** 10.1038/s41598-017-00623-9

**Published:** 2017-04-03

**Authors:** R. Kobayashi, T. Kobayashi, F. Sakai, T. Hosoya, M. Yamamoto, T. Kurita-Ochiai

**Affiliations:** 1 Department of Microbiology and Immunology, Nihon University School of Dentistry at Matsudo, Chiba, 271-8587 Japan; 2 Public Relations Department, Megmilk Snow Brand Co., Ltd, 13, Honshiocho, Shinjuku-ku, Tokyo 160-8575 Japan; 3 Milk Science Research Institute, Megmilk Snow Brand Co., Ltd, 1-1-2, Minamidai, Kawagoe, Saitama 350-1165 Japan

## Abstract

Probiotics have been used to treat gastrointestinal disorders. However, the effect of orally intubated probiotics on oral disease remains unclear. We assessed the potential of oral administration of *Lactobacillus gasseri* SBT2055 (LG2055) for *Porphyromonas gingivalis* infection. LG2055 treatment significantly reduced alveolar bone loss, detachment and disorganization of the periodontal ligament, and bacterial colonization by subsequent *P*. *gingivalis* challenge. Furthermore, the expression and secretion of TNF-α and IL-6 in gingival tissue was significantly decreased in LG2055-administered mice after bacterial infection. Conversely, mouse β-defensin-14 (mBD-14) mRNA and its peptide products were significantly increased in distant mucosal components as well as the intestinal tract to which LG2055 was introduced. Moreover, IL-1β and TNF-α production from THP-1 monocytes stimulated with *P*. *gingivalis* antigen was significantly reduced by the addition of human β-defensin-3. These results suggest that gastrically administered LG2055 can enhance immunoregulation followed by periodontitis prevention in oral mucosa via the gut immune system; i.e., the possibility of homing in innate immunity.

## Introduction

*Porphyromonas gingivalis*, a Gram-negative anaerobe, is one of the major pathogens associated with chronic periodontitis, a disease that causes the destruction of alveolar bone, and, as a consequence, tooth loss^1^. Recent evidence suggests that this bacterium contributes to periodontitis by functioning as a keystone pathogen^2, 3^. Virulence factors of *P*. *gingivalis*—including lipopolysaccharide (LPS), hemagglutinins, gingipains, and fimbriae—are important in the induction of immune inflammatory responses and alveolar bone resorption^4–7^. Furthermore, it has been suggested that chronic inflammation caused by periodontopathic bacteria influences systemic diseases such as cardiovascular diseases, diabetes, respiratory diseases, and low-birth-weight infants^8^. Therefore, the prevention of periodontal inflammation may be useful for the prevention of pathogen-associated systemic diseases.

The effect of *Lactobacillus* on human health has been examined for many years. Numerous studies have confirmed the beneficial activity of some exogenous lactic acid bacteria in the treatment and prevention of rotaviral infection, antibiotic-associated diarrhea, inflammatory bowel disease, and other gastrointestinal disorders^9^.

*Lactobacillus gasseri* is an indigenous bacterium that colonizes the gastrointestinal tract, oral cavity, and vagina of humans^10^. *L. gasseri* elicits various health benefits through antimicrobial activity, bacteriocin production, and immunomodulation of the innate and systemic immune responses^11^. *Lactobacillus gasseri* SBT2055 (LG2055) is a probiotic lactic acid bacterium with properties such as bile tolerance and the ability to improve the intestinal environment^12–15^. Increasing evidence also suggests that the induction of epithelial signaling by intestinal lactobacilli can modulate barrier functions and defensin production, and regulate inflammatory signaling^16^. As an important member of the defensin family, mouse β-defensin-14 (mBD14), an antimicrobial ortholog of human β-defensin-3, can contribute to the local innate immune response by combating microbial invasion^17^. Although β-defensin plays a crucial role in the anti-infectious response at local sites^18^, its effects on the inflammatory response and the possible mechanism in the mouth and at remote mucosal sites remain unknown. Therefore, the present study examined whether oral administration of *Lactobacillus gasseri* SBT2055 is effective for preventing experimental periodontal disease.

## Results

### Gastric intubation of LG2055 suppresses alveolar bone loss, detachment and disorganization of the periodontal ligament in mice infected with *P*. *gingivalis*

**Figure 1:**
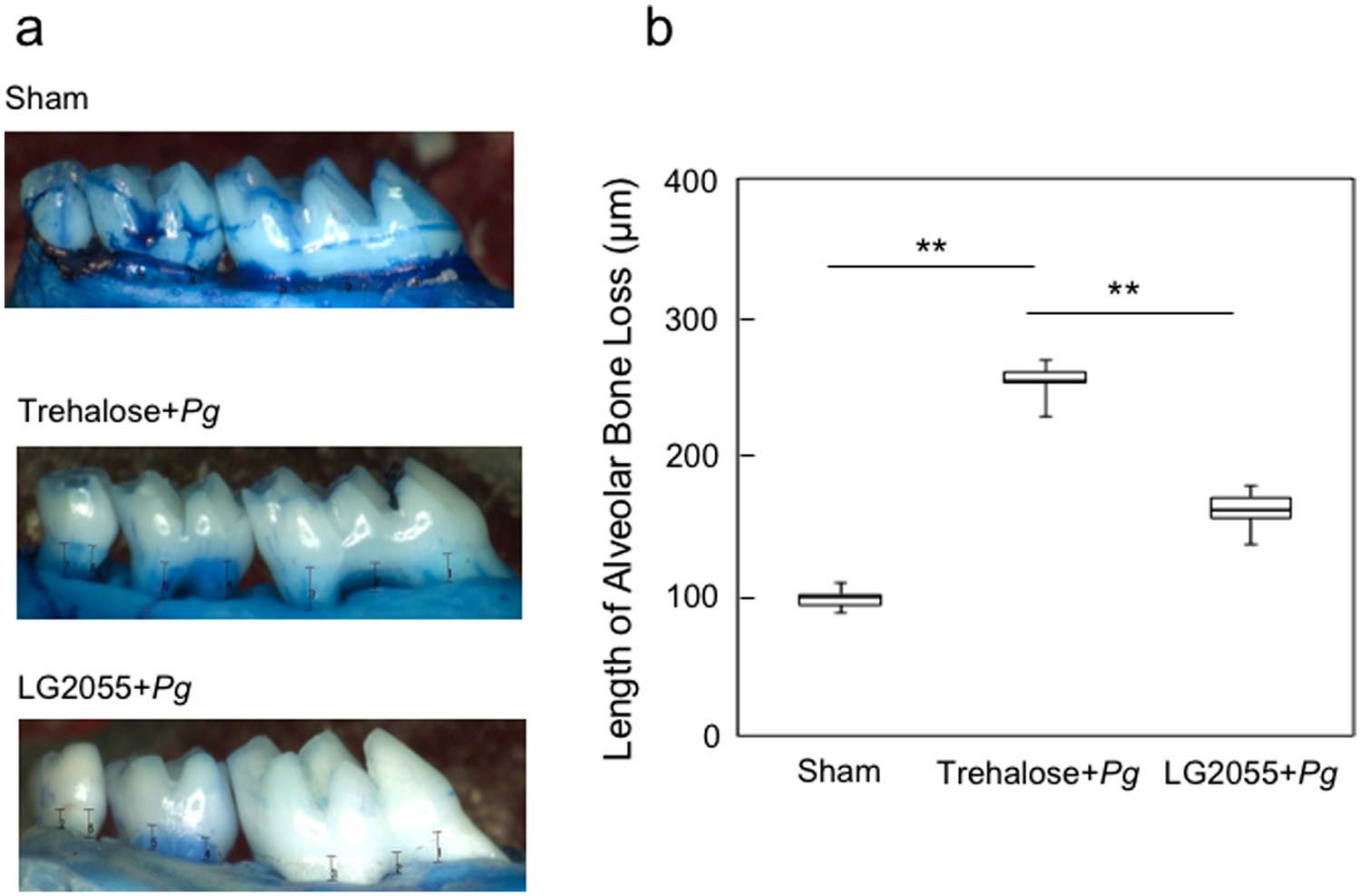
Reduction in *P*. *gingivalis*-induced alveolar bone loss by gastric intubation with LG2055. (**a**) Thirty days after *P*. *gingivalis* infection, the distance from the cementoenamel junction (CEJ) to the alveolar bone crest (ABC) at 14 predetermined sites in the defleshed maxilla were measured and totaled for each mouse. (**b**) Bone measurements were performed a total of three times by two evaluators using a random and blinded protocol. All values are presented as the means ± SEM of eight mice per group; **p < 0.01.

**Figure 2:**
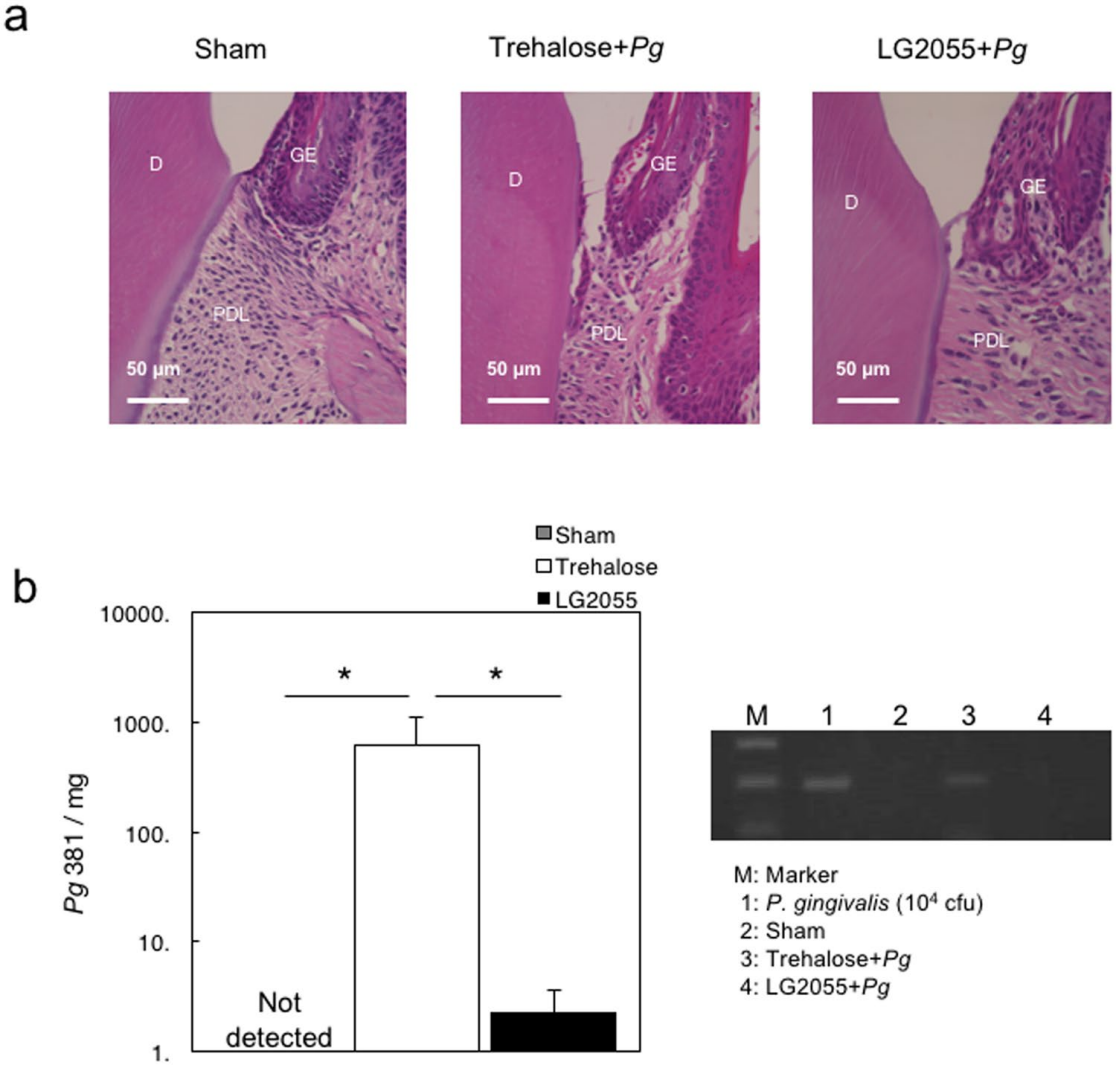
Suppression of *P*. *gingivalis*-induced detachment and disorganization of the periodontal ligament, and bacteria detection by gastric intubation with LG2055. (**a**) Histochemical analysis of gingival tissue. Thirty days after *P*. *gingivalis* infection, mouse lower jaws with gingival tissue were stained with hematoxylin and eosin. D: dentin, GE: gingival epithelium, PDL: periodontal ligament. (**b**) Detection of *P*. *gingivalis*-specific 16 S rRNA. Thirty days after *P*. *gingivalis* infection, DNA was extracted from gingival tissues of mice and amplified using real-time quantitative PCR with a pair of primers corresponding to *P*. *gingivalis*-specific 16 S rRNA. Different numbers of DNA from *P*. *gingivalis* 381 were used to generate a standard curve. All values are expressed as the means ± SEM per mg of tissue for eight mice per group; *p < 0.05.

To examine the effect of gastric intubation of LG2055 on the prevention of alveolar bone loss and gingival inflammation induced by *P*. *gingivalis*, mice administered LG2055 or 25% trehalose alone were infected orally with *P*. *gingivalis*. Mice gastrically intubated with LG2055 showed a significant reduction in alveolar bone loss caused by *P*. *gingivalis* infection compared to the loss in trehalose-treated mice (Fig. 1). Detachment and disorganization of the periodontal ligament were also reduced in mice gastrically intubated with LG2055 followed by oral infection with *P*. *gingivalis* (Fig. 2a). Furthermore, *P*. *gingivalis*-specific 16S rRNA isolated from gingival tissue 30 days after infection, which is equivalent to that isolated from approximately 1 × 10^4^ *P*. *gingivalis*, was markedly reduced by gastric intubation of LG2055 (Fig. 2b). Notably, no LG2055-specific DNA was detected in gingival tissue 30 days after the last LG2055 treatment.

### Gastric intubation of LG2055 reduces the inflammatory response in gingival mononuclear cells (GMCs) and gingival biopsies

**Figure 3:**
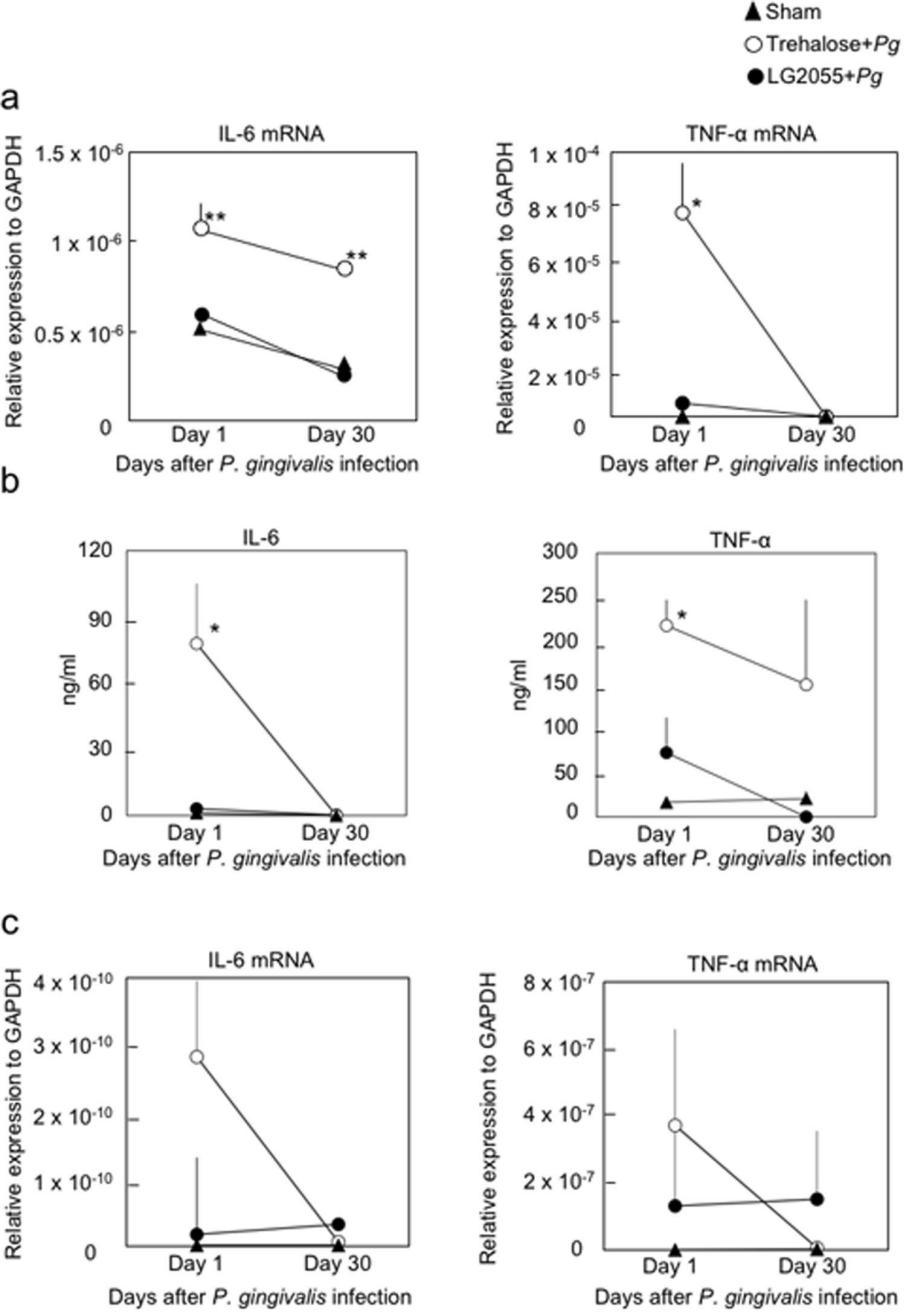
Suppression of the *P*. *gingivalis*-induced inflammatory cytokine response in gingival tissues and GMCs by gastric intubation with LG2055. (**a**) Total RNA was extracted from gingival tissue 1 and 30 days after infection with *P*. *gingivalis*, and IL-6 and TNF-α mRNA levels were determined using real-time PCR. (**b,c**) GMCs (1 × 10^6^/mL) were isolated 1 and 30 days after *P*. *gingivalis* infection, and then cultured for 3 days. (**b**) The culture supernatants were collected and subjected to IL-6- and TNF-α-specific ELISAs. (**c**) The cultured cells were harvested for RNA isolation and quantification of IL-6 and TNF-α mRNA using real-time PCR. All values are presented as the means ± SEM of eight mice per group at each time point; **p < 0.01, *p < 0.05.

To examine the effect of LG2055 on inflammatory responses in gingival tissues of mice orally administered *P*. *gingivalis*, we examined the IL-6 and TNF-α mRNA levels in gingival tissues and GMCs, and IL-6 and TNF-α secretion in GMCs 1 and 30 days after the final infection. *P*. *gingivalis*-infected mice produced higher amounts of IL-6 and TNF-α mRNA in the gingival tissues and GMCs, and higher protein levels in GMCs than those in sham-infected mice 1 day after the final infection. In contrast, the IL-6 and TNF-α mRNA and protein levels following *P*. *gingivalis* infection were significantly reduced by the oral administration of LG2055 before infection (Fig. 3a,b and c). These results may reflect differences in cell populations producing inflammatory cytokines. Our results show that LG2055 administration led to decreased proportions of CD3^+^ and B220^+^ cells and increased proportions of CD11b^+^ and CD11c^+^ cells among GMCs (Supplementary Table S1). In future, it may be necessary to identify the cells in GMC-enriched populations that produce IL-6 and TNF-α in response to *P*. *gingivalis* stimulation.

### Mouse β-defensin-14 induction in gingival tissues by gastric intubation of LG2055

**Figure 4:**
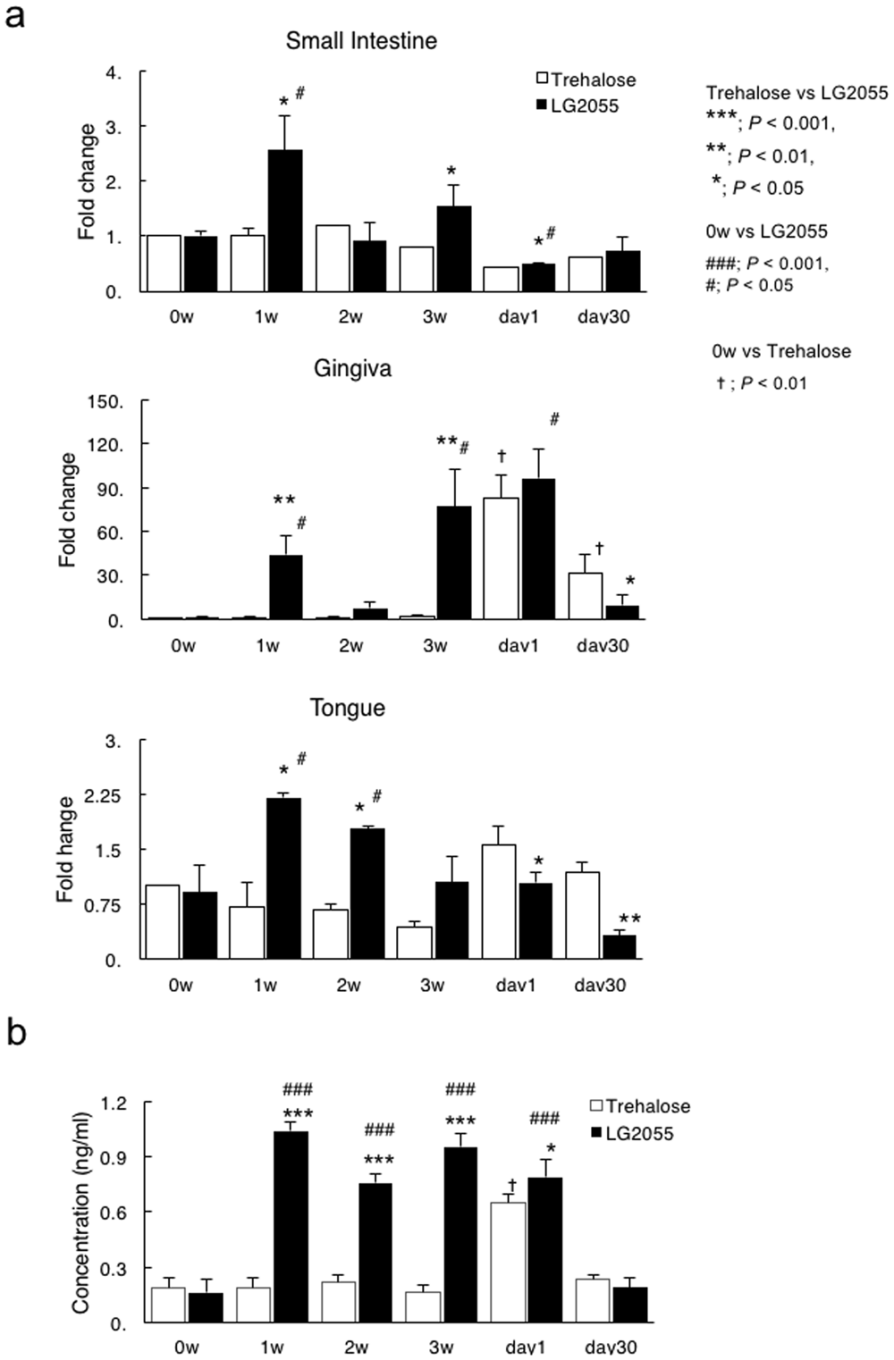
β-Defensin-14 expression and protein production in mucosal components of mice administered LG2055 (**a,b**). At 0–3 weeks after LG2055 administration and 1 and 30 days after the final *P*. *gingivalis* infection, total RNA was extracted from gingival tissue, tongue, and small intestine, and mRNA levels were determined using quantitative real-time PCR (**a**). Similarly, saliva samples were collected and subjected to a β-defensin-3-specific ELISA (**b**). All values are presented as the means ± SEM of five mice per group at each time point; **p < 0.01, *p < 0.05.

The role of antimicrobial peptides may be especially important for the oral cavity as it is constantly exposed to microbial challenges. Furthermore, it was shown that defensins could suppress early events in inflammation and enhance systemic antibody responses^18^. Since several reports have indicated that lactobacilli enhance the production of antimicrobial peptides in mucosal surfaces such as the gut^19^, we examined whether mBD14 could be induced in distant mucosal surfaces (such as gingival tissues) of mice gastrically intubated with LG2055. One week after the onset of gastric intubation with LG2055, significant expression of mBD14-specific mRNA was detected in the small intestine, gingival tissue, and tongue (Fig. 4a). Notably, mBD14-specific mRNA levels were significantly induced in gingival tissue and tongue compared with the small intestine 2 weeks after LG2055 administration, and those levels were maintained until the first infection with *P*. *gingivalis* (Fig. 4a). Furthermore, the production of mBD14 was significantly enhanced in saliva of mice 3 weeks after gastric intubation with LG2055 (Fig. 4b). These results suggest that LG2055 administration could enhance the induction of mBD14 in not only the small intestine but also the oral cavity. The results also raise the possibility that an increase in β-defensin levels in the gingiva and saliva after LG2055 administration suppressed inflammatory cytokine production in response to *P*. *gingivalis* infection (Figs 3 and 4). On the other hand, in the trehalose group, no increase in β-defensin levels was detected before *P*. *gingivalis* infection. These results suggest that it is important to increase β-defensin production before exposure for successful infection prevention.

### Human β-defensin-3 suppresses inflammatory cytokine production in monocytes

**Figure 5:**
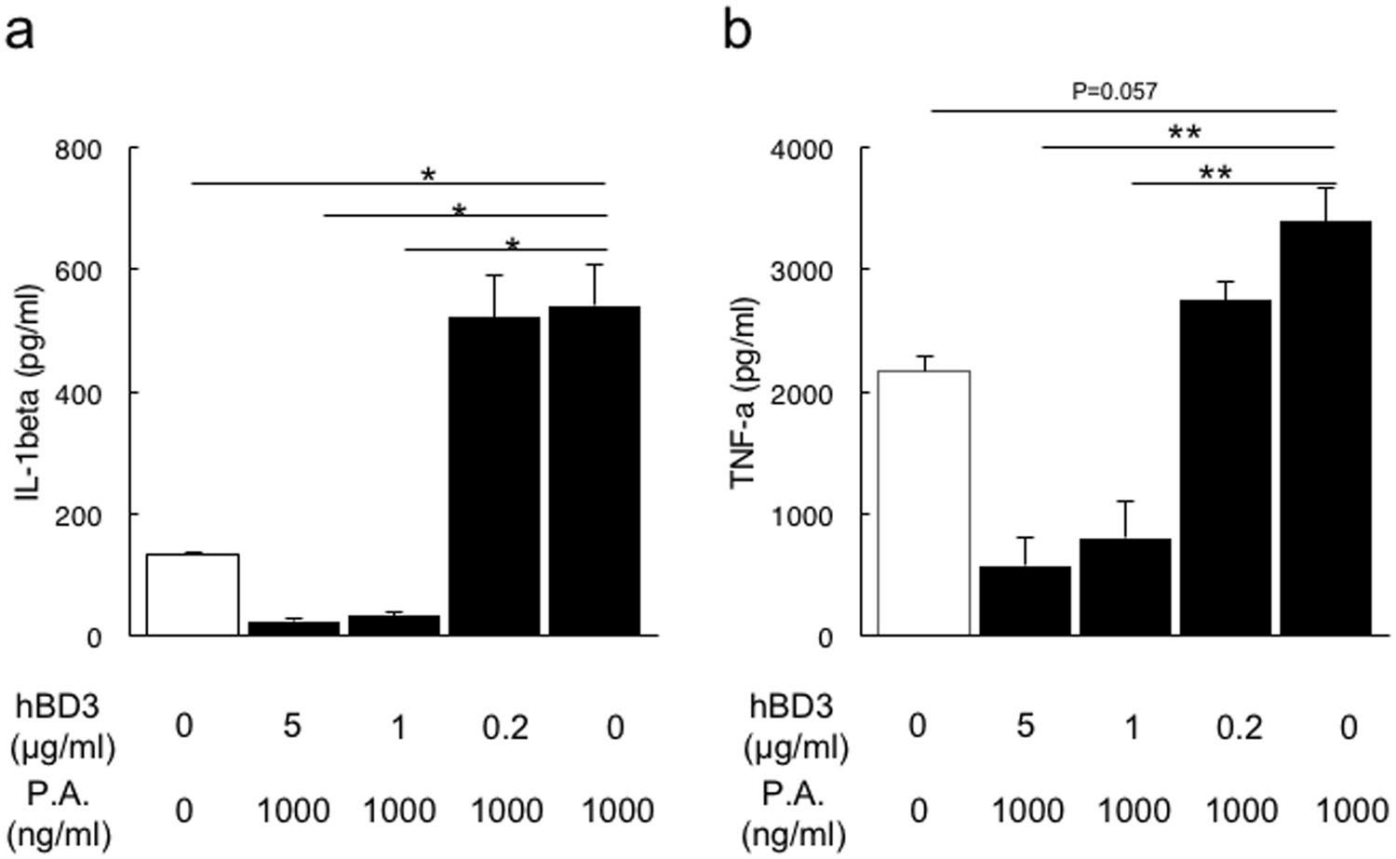
Suppression of *P*. *gingivalis* antigen-induced inflammatory cytokine release in THP-1 by hBD3. PMA-primed THP-1 cells were pretreated with various doses of hBD3 for 30 min and stimulated with 1,000 ng/mL of *P*. *gingivalis* antigen for 23 h. The culture supernatants were collected and subjected to IL-1β- and TNF-α-specific ELISAs. The data are presented as the means SD (n = 3) of three independent experiments; **p < 0.01, *p < 0.05.

To examine the direct effect of β-defensin on inflammatory cytokine production, we assessed the levels of IL-1β and TNF-α in supernatants prepared from THP-1 cultures treated with *P*. *gingivalis* antigen for 23 h. The supernatants of THP-1 cultures treated with 1,000 ng/mL of *P*. *gingivalis* antigen exhibited higher levels of IL-1β (p < 0.05) and TNF-α (p = 0.057) compared with the controls (Fig. 5). In contrast, the addition of recombinant human β-defensin-3 (rhBD-3) (≥1 μg/mL) to the culture significantly suppressed IL-1β and TNF-α production. These findings suggest that β-defensin directly inhibits the inflammatory cytokine production caused by *P*. *gingivalis* infection.

## Discussion

The present study demonstrates the protective effects of gastric intubation of *Lactobacillus gasseri* SBT2055 (LG2055) against *P*. *gingivalis* oral infection. The effects allow mice to be resistant to bacterial infection, as shown by the improvement in bone resorption (Fig. 1), improvement in detachment and disorganization of the periodontal ligament (Fig. 2a), and the decrease in bacteria (Fig. 2b). It has been reported that massive inflammatory cell infiltration into gingival tissues and excessive proinflammatory cytokine production frequently occur during the course of periodontal infection. Therefore, we evaluated IL-6 and TNF-α mRNA and protein levels in gingival tissues and GMCs. As shown in Fig. 3a, the IL-6 and TNF-α mRNA levels in gingival tissues after *P*. *gingivalis* infection were significantly decreased in the group that received LG2055. Furthermore, IL-6 and TNF-α mRNA and protein levels were also lower in the GMCs of LG2055-administered mice compared to those of control mice 1 day after *P*. *gingivalis* infection (Fig. 3b and c). These results suggest that LG2055 has a preventive effect against *P*. *gingivalis*-induced experimental periodontitis by regulating these inflammatory cytokines.

A recent study showed that oral infection with *P*. *gingivalis* induces systemic inflammation and metabolic changes associated with alterations in the gut microbiota^20^. Since β-defensin contributes to homeostatic control of the enteral environment, it is possible that the decrease in defensin induces dysbiosis, disrupts enteral environment, and leads to illness. Clinical studies have linked the defective expression of **β**-defensin to the reduced killing of certain microorganisms by the intestinal mucosa of patients suffering from ileal and colonic Crohn’s disease (CD)^21^. Previous studies have shown that **β**-defensin expression or secretion was significantly upregulated in Caco-2 cells upon stimulation by several *Lactobacillus* species^22^. Because LG2055 is an intestinal commensal bacterium, we examined whether the gastric intubation of LG2055 induced the expression and production of β-defensins in not only the gut but also oral sites. Our results show that the expression of β-defensin, which is critical for antibacterial activity, was enhanced in small intestine, gingival tissue, and tongue 1 week after the administration of LG2055. Moreover, β-defensin was secreted in the saliva more than 3 weeks after LG2055 administration. Since the number of *P*. *gingivalis* in gingival tissue was significantly decreased by the gastric intubation of LG2055, it was suggested that β-defensin production in the oral cavity affects bacterial abundance. We confirmed that the viability of *P*. *gingivalis* was decreased by the addition of β-defensin-3 *in vitro*. The β-defensin levels naturally varied between *in vivo* and *in vitro* conditions, but, as an example, the survival rate of 1 × 10^6^ CFU of *P*. *gingivalis* decreased by 25% with the addition of 10 mg/mL of β-defensin, and by 75% with the addition of 25 mg/mL of β-defensin (unpublished observation).

A similar effect on *P*. *gingivalis* was shown previously^23^. Further, IL-1β and TNF-α production from THP-1 cells in response to *P*. *gingivalis* antigen was significantly suppressed by the addition of hBD3. This suggests that hBD3 could significantly control the production of inflammatory cytokines both by antibacterial activity against *P*. *gingivalis* and by antigen sensitization followed by *P*. *gingivalis* infection. Indeed, β-defensins have powerful anti-inflammatory effects on human monocytes^24^, human monocyte-derived macrophages^25^, and human myeloid dendritic cells (DCs)^26^ treated with LPS or recombinant *P*. *gingivalis* hemagglutinin B. These results suggest that the effect of LG2055 may contribute to enhancement of the host defense system in oral sites prior to periodontopathic bacterial infection. Furthermore, since β-defensin was also secreted in gingival tissue, tongue, and saliva, which are remote organs, it was suggested that intestinal cells sensitized with LG2055 could migrate, act on remote tissue cells, and prompt β-defensin production locally. We cannot explain why β-defensin expression decreased suddenly in week 2 in both the small intestine and gingiva. However, β-defensin expression tended to be maintained in the gingiva and tongue for a longer time whereas expression in the small intestine was transient, and this tendency was consistent aside from during the second week. The durability of β-defensin should be checked in future work.

Recently, it was reported that DCs in the intestinal tract migrate into remote secondary lymphoid organs and influence local sites^27^. Therefore, enteric DCs sensitized with LG2055 may migrate and promote the production of β-defensin by interacting with oral epithelial cells. According to our data, a significant increase in DCs among GMCs was observed after LG2055 sensitization (Supplementary Fig. S1). We plan to confirm whether this phenomenon is specific to LG2055. Previous studies have shown that tumor antigen-pulsed DCs migrate to the upper respiratory tract after mucosal administration in humans^28^. Moreover, cross talk with DCs is required for β-defensin production in gingival epithelial cells^29^. In contrast, it was shown that IL-17 and IL-22 promote β-defensin production in epithelial cells^30, 31^. Furthermore, IL-17-producing γδ T cells could induce CXCL8-mediated migration and IL-17-dependent production of β-defensin by epithelial cells^32^. Intestinal innate lymphoid cells (ILCs), including NK-like cells, lymphoid tissue inducer (LTi) cells, and γδ IELs also respond to pro-inflammatory cytokines to upregulate IL-22. Therefore, enteric IL-17-producing γδ T cells^32, 33^ or IL-22-producing ILCs^34^ induced by LG2055 may migrate and promote the production of β-defensin by interacting with oral epithelial cells.

In conclusion, our results demonstrate that gastric administration of LG2055 could control oral inflammation and bone resorption by *P*. *gingivalis* infection. Furthermore, the suppression of inflammatory cytokine production in gingival tissue by gastric administration of LG2055 may correlate with β-defensin production in oral sites. Since the administration of tablets containing *Lactobacillus reuteri* significantly decreased the number of periodontal pathogens in the subgingival microbiota and was effective as an adjunct to scaling and root planing in chronic periodontitis in a randomized clinical trial^35, 36^, it is probable that the results observed in our animal experiment will also be seen in the human oral cavity in clinical trials. Further study is required regarding the detailed mechanism underlying the induction of β-defensin production in the oral cavity by gastric administration of LG2055.

## Methods

### Mice

Eight-week-old female BALB/c Cr Slc (BALB/c) mice, obtained from Sankyo Laboratories (Tokyo, Japan), were provided regular mouse feed and water *ad libitum*. The mice were maintained under specific-pathogen-free conditions on temperature-controlled clean racks with a 12-h light-dark cycle. All animal experiments were performed in accordance with the guidelines of the Bioscience Committee of Nihon University and were approved by the Institutional Animal Care and Use Committee of Nihon University (Approval number: AP11MD016).

### *Lactobacillus gasseri* SBT2055 (LG2055) preparation

LG2055 was provided by the Milk Science Research Institute, Megmilk Snow Brand Co., Ltd. LG2055 was cultured in MRS broth (Difco Laboratories, Detroit, MI, USA) at 37 °C for 18 h and harvested by centrifugation at 10,000 × *g* for 15 min at 4 °C. The cells were washed twice with sterile PBS (−), resuspended in 25% trehalose solution, and stored at −80 °C until use. The influence on total viable bacterial count by freeze thawing was minimal.

### Bacterial strain

*Porphyromonas gingivalis* strain 381 was cultured anaerobically as described previously^37^. The bacteria were harvested from brain heart infusion broth (Difco Laboratories) supplemented with hemin (5 mg/mL) and menadione (0.4 mg/mL) and resuspended in 5% carboxymethyl cellulose (CMC) for oral infection.

### Experimental design

**Figure 6:**
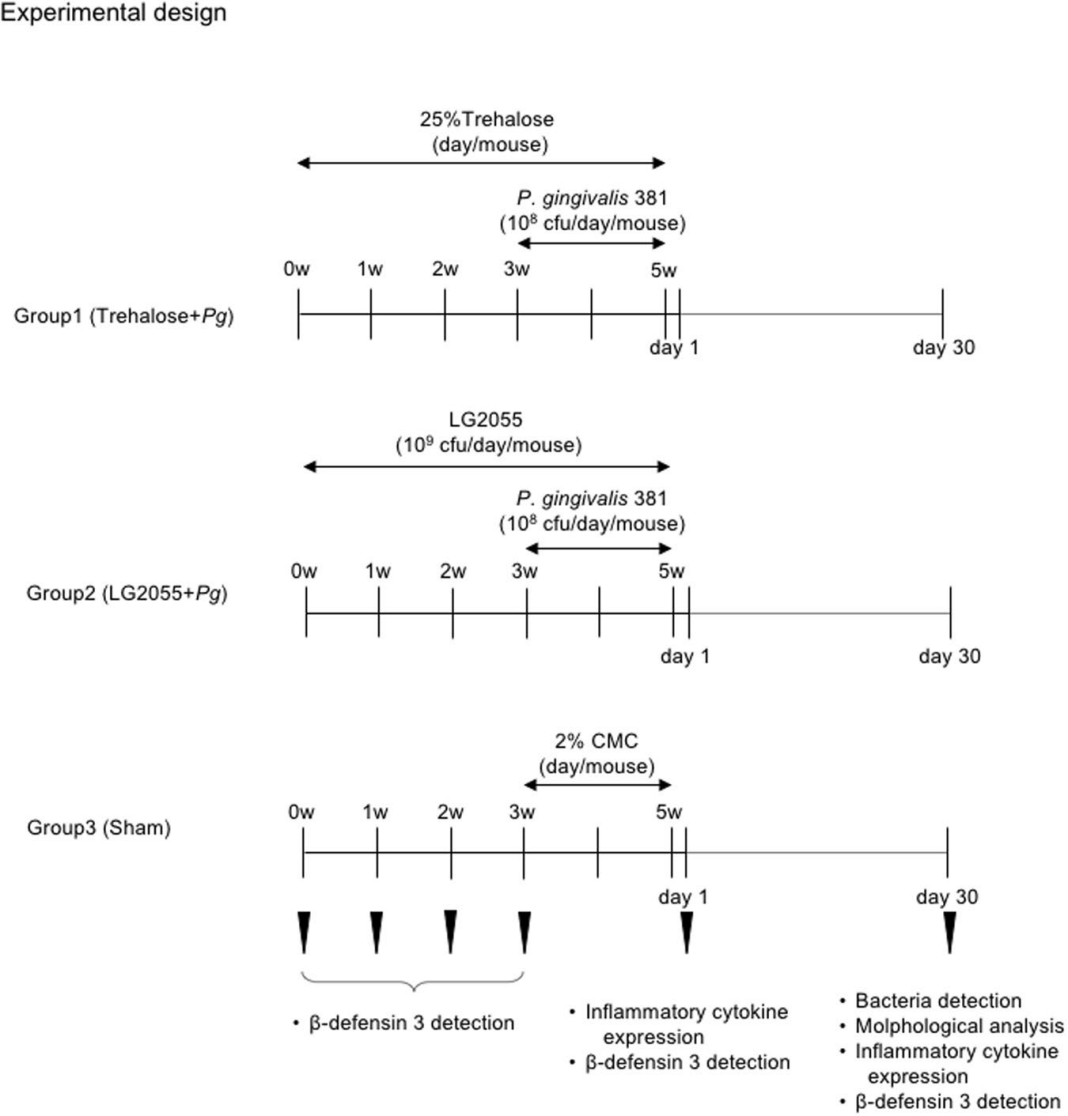
Experimental procedure. Eight-week-old female BALB/c mice were randomly divided into three groups (n = 36 per group); groups 1 and 2 were orally intubated with 25% trehalose or LG2055 (1 × 10^9^ CFU/200 μL/mouse) throughout the experiment once per day for 5 weeks. After administration for 3 weeks, mice were orally infected with *P*. *gingivalis* (1 × 10^8^ CFU/100 μL with 5% CMC/mouse) once per day for 2 weeks. The third group received 5% CMC without oral intubation. Mice were sacrificed 0, 1, 2, and 3 weeks (n = 5 at each time point) after LG2055 administration for a mouse β-defensin-3 assay, and were sacrificed 1 and 30 days (n = 8 at each time point) after *P*. *gingivalis* infection for mouse β-defensin-3 assay, GMC cultures, bacterial detection, histological analysis, and alveolar bone loss evaluation. The English in this document has been checked by at least two professional editors, both native speakers of English. For a certificate, please see: http://www.textcheck.com/certificate/bnmrY9.

Mice were randomly divided into three groups (n = 36 per group; Fig. 6). The first and second groups were orally intubated with a 25% trehalose solution or LG2055 suspension (1 × 10^9^ CFU/200 µL/mouse) through a syringe fitted with a ball-type feeding needle once per day for 5 weeks. At 3 weeks after oral intubation was started, the mice were orally infected with live *P*. *gingivalis* (1 × 10^8^ CFU/100 μL with 5% CMC/mouse) once per day for 14 days. The third group consisted of sham-infected mice that received CMC without oral intubation. LG2055 and *P*. *gingivalis* were administered at specific time intervals.

### Histological analysis of gingival tissue and the measurement of alveolar bone loss

Thirty days after the last infection, mice were sacrificed and the skin of the lower jaw was removed and fixed in 1% paraformaldehyde in PBS for 24 h. The lower jaw was decalcified by incubation in 150 mM EDTA in PBS for 5–7 days at 4 °C and then embedded in paraffin. Four-micrometer-thick serial sections were then prepared and stained with hematoxylin and eosin. Horizontal bone loss around the maxillary molars was assessed using a morphometric method, as described previously^38^. Briefly, after removing gingival tissue, skulls were immersed overnight in 3% hydrogen peroxide, pulsed for 1 min in bleach, and stained with 1% methylene blue. The distance from the cementoenamel junction to the alveolar bone crest was measured at 14 buccal sites per mouse^39^. Measurements were made under a dissecting microscope (×20) fitted with a video image marker measurement system standardized to provide measurements in millimeters.

### GMC isolation

At 1 and 30 days after the final *P*. *gingivalis* infection, mice were sacrificed and gingival tissues from the upper and lower jaws were carefully removed using microsurgical tweezers under a stereomicroscope. Cells from gingival tissues were prepared by gently teasing the tissue through sterile stainless steel screens, followed by an enzymatic dissociation procedure with 0.3 mg/mL of collagenase (Nitta Gelatin Co. Ltd., Osaka, Japan) in RPMI 1640 (Wako Pure Chemical Industries Ltd., Osaka, Japan)^40^. GMCs were enriched to 60–80% purity through discontinuous Percoll gradients (Pharmacia Fine Chemicals, Uppsala, Sweden) and resuspended in RPMI 1640 supplemented with HEPES buffer (15 mM), L-glutamine (2 mM), penicillin (100 U/mL), streptomycin (100 μg/mL), and 10% fetal bovine serum (Biofill, Victoria, Australia) (complete medium). GMC-enriched populations (2 × 10^5^ cells) were stained with a combination of fluorescence-conjugated or biotinylated monoclonal antibodies, including anti-CD3, -B220, -CD11c, and -CD11b (BD Pharmingen, San Diego, CA, USA). The samples were subjected to FACS analysis to confirm cell purity and phenotype. The GMCs contained mononuclear cells positive for CD3 and B220, as well as monocytes (including macrophages and DCs) with >99% viability (Supplementary Table S1).

### Cytokine- and β-defensin-specific ELISAs

GMCs (1 × 10^6^/mL) were cultured in complete medium for 3 days and the culture supernatants were collected and subjected to IL-6- and TNF-α-specific ELISAs. We used a mouse IL-6 and TNF-α immunoassay kit (R&D Systems Inc., Minneapolis, MN, USA) to quantify IL-6 and TNF-α in culture supernatants. After removing the culture supernatant, total RNA was extracted from GMCs and subjected to quantitative real-time PCR for IL-6 and TNF-α mRNA. Salivary β-defensin-3 levels were analyzed using a Mouse DEFB3/Beta Defensin 3 ELISA Kit (LSBio, Seattle, WA, USA). Briefly, mouse saliva was collected 1, 2, and 3 weeks after the initial administration of LG2055, and on days 1 and 30 after the last *P*. *gingivalis* infection. Since β-defensin has a strong positive electric charge, the saliva samples were pretreated with Tween 20 and dilute HCl to break the bond between β-defensin and components with a negative electric charge (e.g., mucin). Briefly, 0.1 N HCl containing 0.5% Tween 20 was added to the saliva samples at a 1:9 ratio and centrifuged at 15,000 × *g* for 10 min at 4 °C. The saliva samples were then stored at −20 °C until analysis.

### Analysis of gene expression in GMCs, gingival tissue, tongue, and small intestine

Every week from 0 to 3 weeks after LG2055 administration and on days 1 and 30 after the final *P*. *gingivalis* infection, total RNA from gingival tissue, tongue, and small intestine samples was extracted using an RNeasy Mini kit and treated with DNase I (Qiagen, Germantown, MD, USA) according to the manufacturer’s instructions. Aliquots of RNA were then reverse-transcribed with oligo(dT) primers using SuperScript^®^ reverse transcriptase (Invitrogen Corp., Tokyo, Japan) to generate cDNA. Quantitative real-time RT-PCR analyses were performed using a Thermal Cycler Dice real-time PCR system (Takara Bio Inc., Otsu, Japan) in accordance with the manufacturer’s protocol. All reactions were carried out in a total volume of 25 mL, containing 30 ng of reverse-transcribed RNA, 12.5 mL of 2x SYBR Green PCR Master Mix (Takara Bio Inc.), and each primer at 100 nM. Specific primers for IL-6, TNF-α, and GAPDH were supplied by Takara Shuzo (Kyoto, Japan). The specific primer for mbD14 was synthesized as described previously^41^. The primer sequences were as follows: IL-6 forward (5′-CCACTTCACAAGTCGGAGGCTTA-3′) and reverse (5′-GCAAGTGCATCATCGTGTTCATAC-3′); TNF-α forward (5′-GGAGTAGACAAGGTAC-3′) and reverse (5′-TATGGCCCAGACCCTCACA-3′); mbD14 forward (5′-TCTTGTTCTTGGTGCCTGCT-3′) and reverse (5′-CGACCGCTATTAGAACATCGAC-3′); and GAPDH forward (5′-TGTGTCCGTCGTGGATCTGA-3′) and reverse (5′-TTGCTGTTGAAGTCGCAGGAG-3′). PCR was performed using the following protocol: 95 °C for 15 min, followed by 40 cycles of 95 °C for 15 s, 60 °C for 10 s, and 72 °C for 30 s. The amplification of each gene and melting curve analysis were performed in triplicate. Target mRNA levels were normalized to that of GAPDH mRNA.

### *Porphyromonas gingivalis*- or LG2055-specific 16 S rRNA

Thirty days after the final *P*. *gingivalis* infection, DNA was extracted from whole gingival tissues from the upper and lower jaws using a QIAamp DNA Mini Kit (Qiagen). Quantification of *P*. *gingivalis* or LG2055 was performed by real-time PCR using *P*. *gingivalis*- or LG2055-specific primers based on 16 S rRNA genes. The primer sequences were as follows: *P*. *gingivalis* forward (5′-AGGCAGCTTGCCATACTGCG-3′) and reverse (5′-ACTGTTAGCAACTACCGATGT-3′); and LG2055 forward (5′-AGCGACCGAGAAGAGAGAGA-3′) and reverse (5′-TGCTATCGCTTCAAGTGCTT-3′). The number of bacteria per weight of the gingival tissues was calculated.

### *In vitro* culture

Acute monocytic leukemia (THP-1) cells were obtained from the JCRB Cell Bank (Health Science Research Resources Bank, Osaka, Japan) and cultured in RPMI 1640 containing 10% fetal bovine serum (Biofill), 10 mM HEPES, 100 μU/mL of penicillin, and 100 μg/mL of streptomycin (Invitrogen Corp.). *Porphyromonas gingivalis* antigen was prepared as described previously^42^. THP-1 cells (1 × 10^6^/well) were pretreated with 0.5 μM PMA for 3 h, and then cultured with *P*. *gingivalis* antigen (1000 ng/mL) for 23 h. To assess the anti-inflammatory effects of β-defensin, cells were pre-incubated for 30 min with 0, 0.2, 1.0, and 5.0 µg/mL doses of recombinant human β-defensin-3 (rhBD-3) before adding antigen. The culture supernatants were then collected and subjected to IL-6- and TNF-α-specific ELISAs (R&D Systems Inc.).

### Statistical analysis

All results are presented as means ± the standard errors of the mean (SEM), and experimental groups were compared with controls using an unpaired non-parametric Mann-Whitney U test in Statview software.

## Electronic supplementary material

Dataset 1
