# Supplementary material for: Oral administration of Lactobacillus gasseri SBT2055 is effective in preventing Porphyromonas gingivalis-accelerated periodontal disease
Source: Sci Rep. 2017 Apr 3;7:545. doi: 10.1038/s41598-017-00623-9 (PMC5428773; doi:10.1038/s41598-017-00623-9)
Supplement: Supplementary file 1 — Dataset 1 [file 41598_2017_623_MOESM1_ESM.doc]

**Oral administration of *Lactobacillus gasseri* SBT2055 is effective in preventing *Porphyromonas gingivalis*-accelerated periodontal disease**

R. Kobayashi, T. Kobayashi, F. Sakai, T. Hosoya, M. Yamamoto and T. Kurita-Ochiai

**Supplementary Figure S1. LG2055 treatment induces CD11c^+^ dendritic cells.** GMC-enriched populations were isolated from gingival tissues. These cells were stained with fluorescent-conjugated anti-CD11c mAbs and subjected to flow cytometry analysis. **, P<0.01 vs Trehalose *, P<0.05 vs Trehalose

Supplementary Figure S1

**Supplementary Table S1. Comparison of the frequencies of CD3^+^, B220^+^, CD11b^+^, and CD11c^+^ cells between groups.**
